# Development of a Novel Chimeric Endolysin, Lys109 With Enhanced Lytic Activity Against *Staphylococcus aureus*

**DOI:** 10.3389/fmicb.2020.615887

**Published:** 2021-01-15

**Authors:** Bokyung Son, Minsuk Kong, Yoona Lee, Sangryeol Ryu

**Affiliations:** ^1^Department of Food and Animal Biotechnology, Seoul National University, Seoul, South Korea; ^2^Department of Agricultural Biotechnology, Seoul National University, Seoul, South Korea; ^3^Department of Food Science and Technology, Seoul National University of Science and Technology, Seoul, South Korea; ^4^Center for Food and Bioconvergence, Research Institute of Agriculture and Life Sciences, Seoul National University, Seoul, South Korea

**Keywords:** *Staphylococcus aureus*, endolysin, domain swapping, screening, antimicrobial agent

## Abstract

As the incidence of antibiotic-resistant bacteria has become increased, phage endolysins are believed as one of the promising alternatives to antibiotics. However, the discovery of potent endolysin is still challenging because it is labor intensive and difficult to obtain a soluble form with high lytic activity. In this respect, the modular structures of Gram-positive endolysins can provide an opportunity to develop novel endolysins by domain rearrangement. In this study, a random domain swapping library of four different endolysins from phages infecting *Staphylococcus aureus* was constructed and screened to obtain engineered endolysins. The novel chimeric endolysin, Lys109 was selected and characterized for its staphylolytic activity. Lys109 exhibited greater bacterial cell lytic activity than its parental endolysins against staphylococcal planktonic cells and biofilms, showing highly improved activity in eliminating *S. aureus* from milk and on the surface of stainless steel. These results demonstrate that a novel chimeric endolysin with higher activity and solubility can be developed by random domain swapping and that this chimeric endolysin has a great potential as an antimicrobial agent.

## Introduction

*Staphylococcus aureus* is a Gram-positive bacterium that threatens human and animal health, causing staphylococcal food poisoning and a wide range of infectious diseases, including skin infections, pneumonia, meningitis, endocarditis, and osteomyelitis (Lowy, 1998; De Lencastre et al., 2007). In particular, the global spread of methicillin-resistant *S. aureus* (MRSA) has raised serious concerns because MRSA can easily become resistant to multiple antibiotics, limiting treatment options (Chambers and Deleo, 2009). Moreover, the strong biofilm-forming ability of *S. aureus* has aggravated problems in the food and medical industries (Lewis, 2001; Otto, 2012). For these reasons, there is an urgent need to create new antimicrobials to combat *S. aureus* (Foster, 2004).

Endolysins are bacteriophage-encoded peptidoglycan hydrolases produced by bacteriophages at the end of their replication cycle to breakdown peptidoglycans of the bacterial cell wall, resulting in the release of viral progeny (Schmelcher et al., 2012a). Endolysins have been suggested as promising antibacterial agents because purified endolysin proteins can rapidly lyse and induce death in Gram-positive bacteria when applied exogenously. Compared to classical antibiotics, endolysins have several advantages because they have narrow host specificity, high sensitivity, and a low probability to develop bacterial resistance (Borysowski et al., 2006). Gram-positive endolysins have a modular architecture with at least two separate functional regions. Generally, the N-terminal domain carries more than one catalytic domain and is attached to the C-terminal cell wall-binding domain (CBD) by a short linker (Fischetti, 2008; Schmelcher et al., 2012a). The catalytic domain determines the enzymatic activity of the endolysin, whereas the CBD positions the catalytic domain to the peptidoglycan of the target bacteria for efficient lysis by the endolysin (Loessner, 2005; Schmelcher et al., 2011).

Most endolysins of staphylococcal phages have three distinct domains: an N-terminal cysteine- and histidine-dependent amidohydrolase/peptidase (CHAP) domain, a central N-acetylmuramoyl-l-alanine amidase (Ami_2 or Ami_3) domain, and a C-terminal SH3b domain as a CBD (Chang and Ryu, 2017). The efficacy of *S. aureus* phage endolysins killing *S. aureus* and controlling staphylococcal infection in animal models has been reported in several studies (Kerr et al., 2001; Rashel et al., 2007; Fenton et al., 2010; Gu et al., 2011). Although several *S. aureus* phage endolysins have presented promising results, poor expression levels and/or insolubility of the expressed proteins have limited the development of highly active staphylococcus-specific phage endolysins (Daniel et al., 2010). In addition, identifying a novel endolysin from *S. aureus* phages is relatively difficult because most *S. aureus*-targeting endolysins have similar domain compositions and display high amino acid sequence identity (Becker et al., 2009b; Oliveira et al., 2013; Chang and Ryu, 2017). To circumvent these problems, a number of research groups have designed truncated or chimeric versions of lysins (Manoharadas et al., 2009; Idelevich et al., 2011; Fernandes et al., 2012), but these trial-and-error strategies are time-consuming and labor-intensive in the search to find a novel endolysin with the desired properties. The modular structure of the functional domains of Gram-positive endolysin allows us to engineer endolysins through domain swapping to generate chimeric endolysins with superior properties (Diaz et al., 1990; Schmelcher et al., 2010). Endolysin engineering such as rationally designed domain recombination or random domain swapping has been endeavored. For example, Yang et al. reported an improved screening of a random domain recombination library of endolysins using controlled lysis of *E. coli* (Yang et al., 2015).

In this study, we developed an induced lysis-based screening to improve the screening efficiency further, enabling us to identify nineteen new chimeras containing different combinations of catalytic and cell wall binding domains from four *S. aureus* phage endolysins. Among them, a novel chimeric endolysin, Lys109, which showed enhanced lytic activity against *S. aureus* and other multiple staphylococcal species, was selected and characterized. This proof-of-concept study confirms the potential of the random domain swapping method to develop a novel therapeutic agent to control *S. aureus*.

## Materials and Methods

### Bacterial Strains and Growth Conditions

The bacterial strains used in this study are listed in Table 1. Staphylococcal strains were grown in tryptic soy broth (TSB) (Difco, Detroit, MI, United States) at 37°C under aerobic conditions. Baird-Parker agar plates with egg yolk tellurite (BPA; Difco) were used for the selective enumeration of *S. aureus*. *Bacillus cereus, B. subtilis, Listeria monocytogenes*, and *Streptococcus thermophilus* was cultivated in brain heart infusion (BHI) medium (Difco). Luria-Bertani (LB) medium (Difco) was used for the growth of Gram-negative strains. *Escherichia coli* DH5α and BL21 (DE3) star strains were used to clone and express proteins, respectively.

**TABLE 1:** Antimicrobial spectrum of LysSA12 and Lys109.

Species	Strains	Antimicrobial activity ^*a*^			
		LysSA12 (pmol)		Lys109 (pmol)	
		167	16.7	167	16.7
*S. aureus*	Human isolate 117	**+**	**–**	**++**	**+**
	Human isolate 119	**+**	**–**	**++**	**–**
	Plant isolate 37	**+**	**–**	**++**	**–**
	Plant isolate 8	**+**	**–**	**++**	**–**
	Plant isolate 13	**+**	**–**	**+**	**–**
	Animal isolate 154	**+**	**–**	**++**	**–**
	Animal isolate 134	**+**	**–**	**++**	**–**
	Animal isolate 100	**+**	**–**	**++**	**–**
	Animal isolate 99	**+**	**–**	**++**	**–**
	Clinical isolate 1163	**+**	**–**	**++**	**–**
	Clinical isolate FMB1	**+**	**–**	**++**	**–**
	Clinical isolate FMB2	**+**	**–**	**++**	**–**
	Clinical isolate FMB3	**+**	**–**	**++**	**–**
	Mastitis cow milk isolate FMB4	**++**	**–**	**++**	**+**
	ATCC 6538	**+**	**–**	**++**	**–**
	RN4220	**+**	**–**	**+ +**	**–**
	ATCC 23235	**+**	**–**	**++**	**+**
	ATCC 13301	**+**	**–**	**+ +**	**–**
	CCARM 3090	**+**	**–**	**+ +**	**–**
*S. hominis*	ATCC 37844	**+**	**–**	**+ +**	**+**
*S. saprophyticus*	ATCC 15305	**+**	**–**	**+ +**	**+**
*S. haemolyticus*	ATCC 29970	**+**	**–**	**+ +**	**–**
*S. capitis*	ATCC 35661	**+**	**–**	**+ +**	**–**
*S. warneri*	ATCC 10209	**+**	**–**	**+**	**–**
*S. xylosus*	ATCC29971	**+**	**–**	**+**	**–**
*S. epidermidis*	CCARM 3787	**+ +**	**+**	**+ +**	**+ +**
*Bacillus cereus*	KCCM 40133	**–**	**–**	**–**	**–**
*B. subtilis*	168	**–**	**–**	**–**	**–**
*Streptococcus thermophilus*	ATCC 19258	**–**	**–**	**–**	**–**
*Listeria monocytogenes*	ATCC 19114	**–**	**–**	**–**	**–**
*Salmonella Typhimurium*	LT2	**–**	**–**	**–**	**–**
*Pseudomonas aeruginosa*	ATCC 27853	**–**	**–**	**–**	**–**
*Cronobacter sakazakii*	ATCC 29544	**–**	**–**	**–**	**–**
*Escherichia coli*	MG1655	**–**	**–**	**–**	**–**

*^a^; ++ clear lysis zone, + turbid lysis zone, **–** no lysis zone.*

### Library Construction for Random Domain Swapping

The gene encoding SPN1S lysRz (SPN1S_0028 and SPN1S_0029) was amplified from *Salmonella* Typhimurium phage SPN1S (GenBank accession number NC_016761) using the oligonucleotides listed in Supplementary Table S1. The gene fragment was digested with *Eco*RI-*Sal*I and inserted into the pBAD33 vector (Lim et al., 2012). To confirm the cell lysis efficiency, the pET28a_EGFP vector (Kong and Ryu, 2015) was cotransformed into competent *E. coli* BL21 (DE3) with pBAD33_SPN1S lysRz. The fluorescence of the released EGFP was measured by using a SpectraMax i3 multimode microplate reader (Molecular Devices, Sunnyvale, CA, United States) with excitation at 485 nm and emission at 535 nm. Four different *S. aureus* phage endolysins, including LysSA12, LysSA97, LysSA11, and LysSAP4, were used to construct two types of libraries (Chang and Ryu, 2017; Chang et al., 2017a). For the first library, we tried to generate chimeric endolysins containing two catalytic domains. The genes encoding four CHAP domains and those encoding three amidase domains from the four endolysins were amplified and digested with *Bam*HI/*Xho*I for insertion as the N-terminal domain of the chimeric endolysins. For the central domain of chimeric endolysins, the seven genes were amplified and digested with *Xho*I/*Bam*HI. All plasmids and primers used in this study are listed in Supplementary Table S1. For the second library, all genes encoding CHAPs and amidase domains were amplified and digested with only *Bam*HI to ensure diversity in the number of catalytic domains to be inserted into the endolysins. All gene fragments were randomly ligated into the *Bam*HI sites of pET28a vectors (Novagen, Madison, WI, United States) containing one of the genes encoding four different cell wall binding domains from the four endolysins. The vector libraries were cotransformed into competent *E. coli* BL21 (DE3) harboring pBAD33_SPN1S lysRz.

### Screening of the Chimeric Endolysins by the Plate Lysis Method

The resulting clones from the random library were screened as described in a previous study with some modifications (Yang et al., 2015). In brief, the clones were picked and grown in 96-well plates with fresh LB broth containing 0.01 mM Isopropyl β-D-1-thiogalactopyranoside (IPTG) and grown overnight at 37°C to initiate the expression of the chimeric endolysins. Afterward, 0.2% arabinose was added to the wells to express SPN1S lysRz. These cultures were dotted onto agar plates overlaid with autoclaved *S. aureus* RN 4220 and incubated for 12 h at 37°C. The clones exhibiting a clear lysis zone against *S. aureus* were screened and picked for sequencing analysis to identify the cloned chimeric endolysin.

### Expression and Purification of the Endolysins

*Escherichia coli* BL21 (DE3) harboring chimeric endolysins were cultivated at 37°C to an optical density at 600 nm (OD_600_) of 0.7 and the protein expression was induced by addition of 0.5 mM IPTG for 20 h at 18°C. Bacterial cells were suspended in lysis buffer (50 mM sodium phosphate, 300 mM sodium chloride and 30% glycerol; pH 8.0) and disrupted by sonication at a duty cycle of 25% and output control of 5 (Branson Ultrasonics, Danbury, CT, United States). After centrifugation (20,000 × *g*, 30 min), the supernatant was passed through a Ni-NTA superflow column (Qiagen GmbH, Hilden, Germany), and purification of the recombinant proteins was performed according to the manufacturer’s instructions. The purified protein was stored at −80°C until use after the buffer was changed to storage buffer (50 mM sodium phosphate, 300 mM NaCl and 30% glycerol; pH 8.0) using PD Miditrap G-25 (GE Healthcare, Amersham, Bucks, United Kingdom).

### Lytic Activity Assay

The lytic activity of the chimeric endolysins and their original endolysins was assessed with a turbidity reduction assay (Son et al., 2012). Bacterial cells grown to the exponential phase were resuspended in reaction buffer (50 mM Tris–HCl, pH 6.5). Then, the purified proteins were added to the cell suspension at a final concentration of 300 nM, and the OD reduction of the cells was measured over time by using a SpectraMax i3 multimode microplate reader at 600 nm. The relative lytic activity was calculated after 60 min as follows: [ΔOD_600_ test (endolysin added) - ΔOD_600_ control (buffer only)]/initial OD_600_. The antimicrobial spectrum was tested by a plate lysis assay as previously described (Chang et al., 2017a). In brief, 10 μL of diluted endolysin (167, 16.7, and 1.67 pmol) was spotted onto a freshly prepared bacterial lawn on TSA agar plates. Spotted plates were air-dried in a laminar flow hood for 15 min and incubated overnight at 37°C. The MIC of the endolysins was determined by serial dilution of the endolysins by 1:2 in 96-well plates as described previously (Andrews, 2001; Swift et al., 2015). Exponentially growing *S. aureus* CCARM 3090 was added to each well at a final concentration of 10^5^ CFU/well, and the plate was incubated at 37°C for 20 h. The MIC was defined as the lowest concentration of endolysin that produced inhibition of visible growth.

### Biofilm Reduction Assay

The biofilm reduction assay was performed as previously described with some modifications (Wu et al., 2003). Staphylococcal strains incubated in TSB medium supplemented with 0.25% D-glucose (Sigma-Aldrich, St. Louis, MO, United States) were prepared and subcultured in the same media in a 96-well polystyrene microplate. After incubating the microplate for 24 h at 37°C, all wells were washed with PBS. Once the biofilm formed, the experimental group wells were filled with endolysins. After incubation for 2 h at 37°C, each well was washed once with PBS and stained with 1.0% crystal violet. Next, each well was washed three times with PBS, followed by solubilization with 33% acetic acid. The absorbance of the obtained solution was measured at 570 nm, and the sessile biomass was presented as an A_570_ value.

### Effect of pH and Temperature on Endolysin Activity

For the temperature stability assay of Lys109, the lytic activity was measured in reaction buffer at 25°C for 60 min after the enzyme was incubated at various temperatures (4–65°C) for 30 min. To study the effects of temperature on Lys109 enzymatic activity, 300 nM Lys109 was added into the target cell suspension, and the mixture was incubated at different temperatures (4–65°C) for 60 min. Then, the lytic activity was measured in reaction buffer at 25°C. To test the effects of pH on the activity of Lys109, 300 nM Lys109 was added to *S. aureus* CCARM 3090 cells suspended in the following buffers: 50 mM sodium acetate (pH 4.5 and 5.4), 50 mM Tris–HCl (pH 6.5–8.0), 50 mM glycine (pH 9.0), and 50 mM N-cyclohexyl-3-aminopropanesulfonic acid (pH 10.0).

### EGFP Fusion Protein Binding Assay

The binding ability of EGFP_LysSA12 amidase plus CBD and EGFP_LysSA97 amidase plus CBD to *S. aureus* cells was evaluated as previously described (Loessner et al., 2002). Bacterial cells grown to the early exponential phase were harvested and resuspended in PBS. EGFP fusion proteins were incubated with the cells for 5 min at 25°C. The mixture was washed twice with PBS to remove unbound protein and was moved to a 96-well plate to measure the fluorescence using a SpectraMax i3 multimode microplate reader (excitation at 485 nm and emission at 535 nm). The OD_600_ of the cells was measured to normalize the fluorescence by calculating the whole-cell fluorescence per OD_600_.

### Antimicrobial Activity Assay in Food Samples

The lytic activity of Lys109 against the MRSA CCARM 3090 strain was tested in commercial whole-fat pasteurized milk as previously described (Chang et al., 2017a). A milk sample was inoculated with exponentially growing MRSA CCARM 3090 cells (approximately 10^5^ CFU/mL). Before the addition of Lys109 and LysSA12 at 0, 30, 300, 900, and 1500 nM, the milk samples were preincubated with the bacteria at 25°C for 1 h to allow the bacteria to adapt to the milk. Each milk sample was then incubated at 25°C for an additional hour. Viable bacterial cells (CFU/mL) were counted every 15 min after the addition of Lys109 and LysSA12 by plating each sample on a BPA plate and incubation at 37°C for 24 h.

### Antimicrobial Activity Assay on Stainless Steel

The lytic activity of Lys109 against the MRSA CCARM 3090 strain was tested on stainless steel as previously described (Cha et al., 2019). A stainless steel coupon with a size of 2 × 2 cm^3^ was sterilized in an autoclave. Exponentially growing MRSA CCARM 3090 bacterial cells were harvested and resuspended in PBS to a final concentration of approximately 10^5^ CFU/mL. Prepared bacterial cells were pipetted onto the stainless steel surface and dried for 1 h on a clean bench. Subsequently, the stainless steel samples were treated with Lys109 (0–100 nM) and left for 60 min at 25°C. For the negative control, PBS was used instead of Lys109 solution. *S. aureus* cells were detached from the surface by agitation in PBST for 2 min with a bench-top vortex mixer at maximum speed. Cell suspensions were serially diluted and plated onto BPA plates and incubated at 37°C for 24 h.

### Statistical Analysis

GraphPad Prism (version 5.01) was used to conduct statistical analysis. One-way analysis of variance (ANOVA) followed by one-way Tukey’s test for all pairwise comparisons (95% confidence interval) was performed. The data are presented as the means with standard deviations. A *P*-value < 0.05 was considered statistically significant.

## Results

### Development of a Random Domain Swapping Method

**FIGURE 1:**
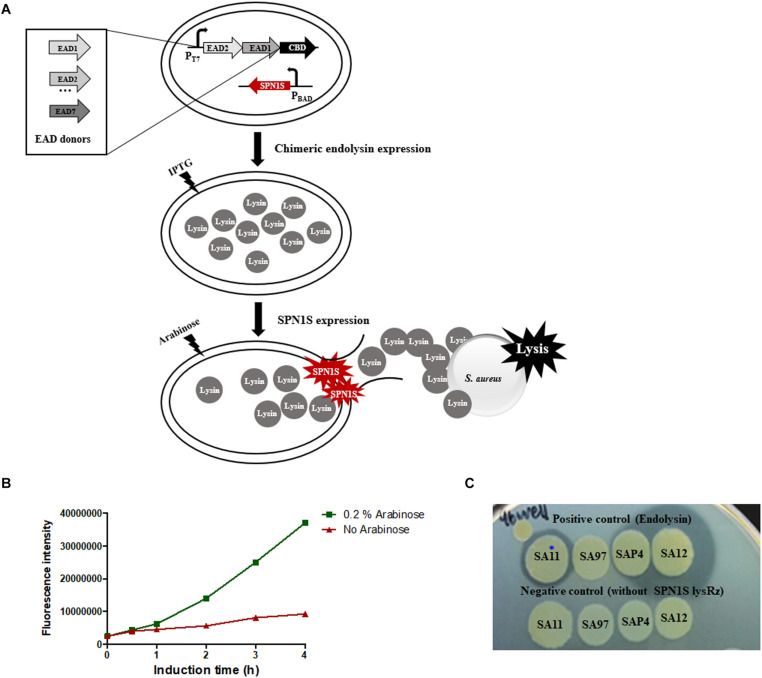
Development of screening system on a 96-well microplate. **(A)** Scheme of the random domain screening method. **(B)** EGFP released from SPN1S-induced lysis in host *E. coli* cells. Cells without induction IPTG for EGFP expression and arabinose for SPN1S lysRz expression) were used as a control. **(C)** Clones for positive and negative controls were cultured with 0.2% arabinose overnight on the lawn of autoclaved *S. aureus* RN4220.

The overall scheme of random domain swapping method was presented in Figure 1A. The protocol was established based on a 96 well plate format for the rapid and efficient screening, taking advantage of a *S.* Typhimurium phage SPN1S lysis cassette composed of an endolysin and Rz/Rz1-like proteins (SPN1S lysRz) (Lim et al., 2012). SPN1S lysRz has been reported to cause host cell lysis and release viral progeny at the end of phage life cycle. To evaluate the cell lysis efficiency of SPN1S lysRz, the amount of EGFP protein released from lysed *E. coli* was measured. Fluorescence significantly increased after induction with arabinose (Figure 1B), indicating that SPN1S lysRz can cause the rapid lysis of *E. coli* cells from within, thereby releasing accumulated proteins in the cytosol. To identify an active chimeric endolysin from a large random library, the lytic efficacy of the released proteins was evaluated by their ability to form a clear zone on an agar plate overlaid with heat-killed *S. aureus* cells. As positive controls, four different *S. aureus* phage endolysins (LysSA11, LysSA97, LysSAP4, and LysSA12) were expressed in the presence of pBAD33_SPN1S lysRz. The clear zones were visualized on the agar plate depending on their activities, whereas clones without pBAD33_SPN1S lysRz did not show a clear zone (Figure 1C). These results suggest that SPN1S lysRz-induced lysis of *E. coli* allowed active chimeric endolysins to form clear zone on agar plates containing target bacteria and that this system can be used as a method for the successful screening of novel chimeric endolysins.

### Isolation of a Novel Endolysin Lys109

**FIGURE 2:**
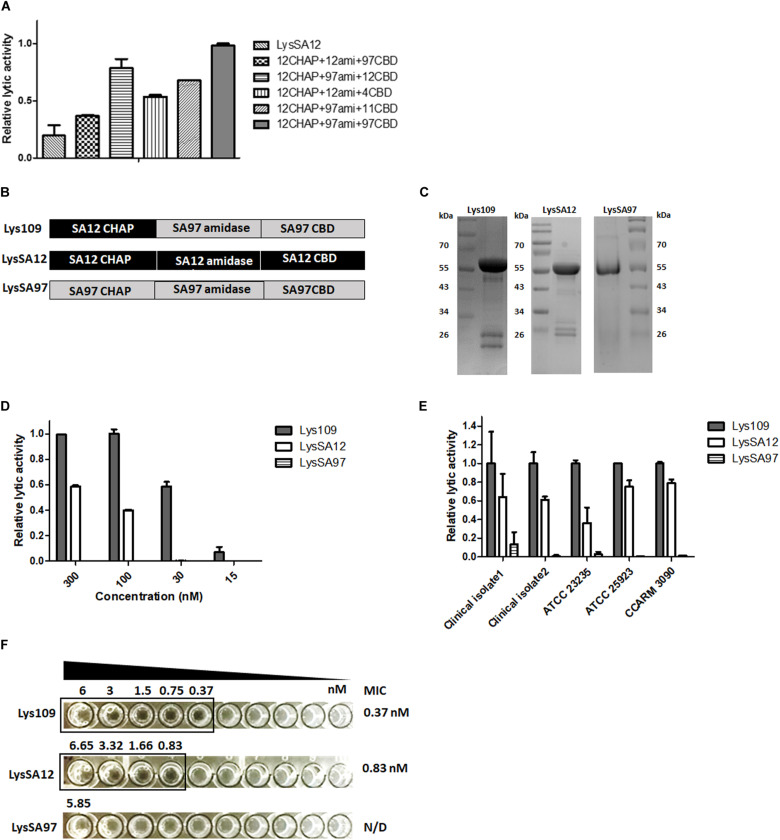
Lytic activity comparison of Lys109 with parental endolysins. **(A)** Comparison of the relative lytic activity of four other chimeric endolysins and LysSA12 with Lys109 (300 nM each) against *S. aureus* CCARM 3090. **(B)** Schematic representation of Lys109, LysSA12 and LysSA97. **(C)** SDS-PAGE analysis of Lys109, LysSA12, and LysSA97. **(D)** Relative lytic activity of Lys109, LysSA12 and LysSA97 against *S. aureus* CCARM 3090 at different concentrations. **(E)** Relative lytic activity of Lys109, LysSA12 and LysSA97 (300 nM each) against various *S. aureus* strains. **(F)** MIC values of Lys109, LysSA12 and LysSA97 against *S. aureus* CCARM 3090.

The random library containing 480 clones were cotransformed into *E. coli* containing the SPN1S lysRz-harboring vector and applied to an agar plate containing *S. aureus* for screening. The clones displaying clear lysis zones were sequenced to determine the combination of the EADs (Supplementary Table S2). Most of the selected clones contained a CHAP domain from LysSA12 or LysSA11, which showed high staphylolytic activity (Chang et al., 2017a, b). In particular, LysSA12 CHAP domain-containing clones, which account for 68% of selected clones, showed a large and clear lysis zone. There was also a single clone containing a LysSAP4 CHAP domain. These results suggest that CHAP domain is necessary to degrade *S. aureus* cell wall peptidoglycan, and this is consistently observed in other chimeric endolysins to control *S. aureus* (Daniel et al., 2010; Schmelcher et al., 2012c; Yang et al., 2014). Among LysSA12 CHAP domain-containing clones, five promising chimeric endolysins were selected for the further comparative analysis (Figure 2A). All selected proteins were expressed in *E. coli* in soluble form and evaluated for their lytic activity against *S. aureus*. As a result, a chimeric endolysin consisting of a LysSA12 CHAP domain in the N-terminal region, a LysSA97 amidase domain at the central and a LysSA97 CBD in the C-terminal region showed the highest lytic activity among the five candidates, and was designated Lys109 (Figure 2B). BLAST analysis revealed that Lys109 has 78% overall amino acid sequence identity with an amidase from *S. aureus* phage StauST398-1 (Van Der Mee-Marquet et al., 2013) and 80% with endolysins from ΦB166 and ΦB236 *S. aureus* phage (Botka et al., 2015). Although the three endolysins showed high similarity with Lys109, they have not yet been studied, suggesting that further research on Lys109 will be meaningful. Moreover, LysSA12, a CHAP domain donor of Lys109, has high amino acid similarity with LysH5 of *S. aureus* phage vB_SauS_phiIPLA88 (98% identity to LysSA12) and *S. aureus* Φ11 endolysin (96% identity to LysSA12) (Sass and Bierbaum, 2007; García et al., 2010). These endolysins have a conserved catalytic triad (C32, H95, and Q112) in their CHAP domains (Supplementary Figure S1). Considering that the lytic activity of *S. aureus* endolysin depends mostly on a CHAP domain, we selected Lys109 to investigate the effect of domain replacement on the activity of the endolysin.

### Lytic Activity of Lys109 in Comparison With Its Parental Endolysins

Lys109 and its parental endolysins, LysSA12 and LysSA97, were highly expressed as a soluble form in *E. coli* and were purified via Ni-NTA affinity chromatography. The predicted molecular weights of Lys109, LysSA12 and LysSA97 were approximately 54 kDa and the proteins migrated as expected in SDS-PAGE gel (Figure 2C). The antibacterial activity of Lys109 was compared with those of LysSA12 and LysSA97 at various concentrations (Figure 2D). Lys109 exhibited clear cell lysis against *S. aureus* CCARM 3090, displaying at least 2-fold higher lytic activity than that of LysSA12 and LysSA97 at all tested concentrations. LysSA12 did not show lytic activity at concentrations below 30 nM and LysSA97 barely exhibited staphylolytic activity at all tested concentrations. These results indicate the superiority of lytic activity of LysSA12 CHAP over LysSA97 CHAP despite their sequence similarity (44% identity). Comparative analysis of the lytic activity of Lys109 with its parental endolysins against other *S. aureus* strains including clinical isolates and MRSA also showed an evident improvement in the lytic activity of Lys109 (Figure 2E). The MIC of Lys109 was compared with those of its parental endolysins. LysSA97, a donor for the amidase domain and CBD of Lys109, did not show inhibition of cell growth at the maximum concentrations available (5.85 μM) (Figure 2F), leading us to exclude LysSA97 in the following experiments. LysSA12 inhibited the growth of *S. aureus* CCARM 3090 at concentration of 0.843 μM. The MIC of Lys109 was 0.375 μM, which was at least 2.25-fold lower than that of LysSA12, indicating that the antimicrobial activity of Lys109 was significantly improved compared to its parental endolysins.

### Biofilm Reduction Activity of Lys109

**FIGURE 3:**
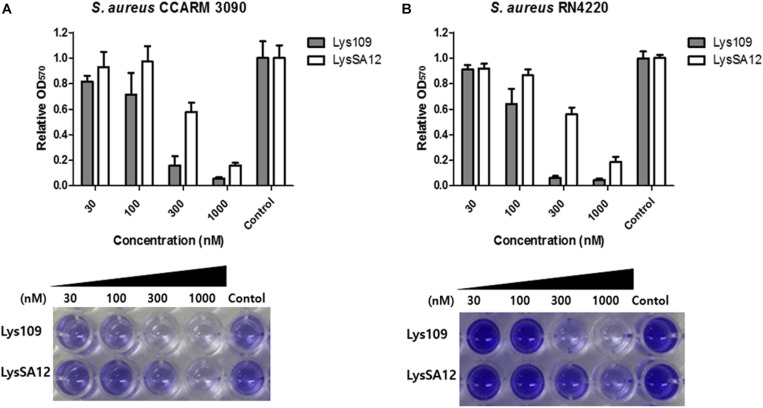
Biofilm reduction activity of Lys109 and LysSA12. Biofilms formed by **(A)** *S. aureus* CCARM 3090 and **(B)** *S. aureus* RN4220 were treated with various concentrations of Lys109 and LysSA12 and visualized by staining biofilms with crystal violet. Dark staining indicates the biofilm that was maintained after treatment with the endolysin. Light or no staining indicates successful removal of the biofilm. Control indicates the sample treated with buffer without endolysins.

The biofilm reduction efficacy of Lys109 against biofilms formed by *S. aureus* CCARM 3090 and *S. aureus* RN4220 was evaluated by a crystal violet staining-based assay (Figure 3). Lys109 showed biofilm reduction activity in a dose-dependent manner, and Lys109 appeared to have a higher biofilm reduction efficacy than LysSA12. When 300 nM of endolysins were added to the biofilms, Lys109 exhibited more than 3-fold enhanced efficacy in removing the biofilms compared to LysSA12. These results demonstrate that Lys109 has strong lytic activity against not only planktonic cells but also biofilms, which is an important contributing factor for many treatment failures (Otto, 2013).

### Temperature and pH Effects on the Enzymatic Activity of Lys109

**FIGURE 4:**
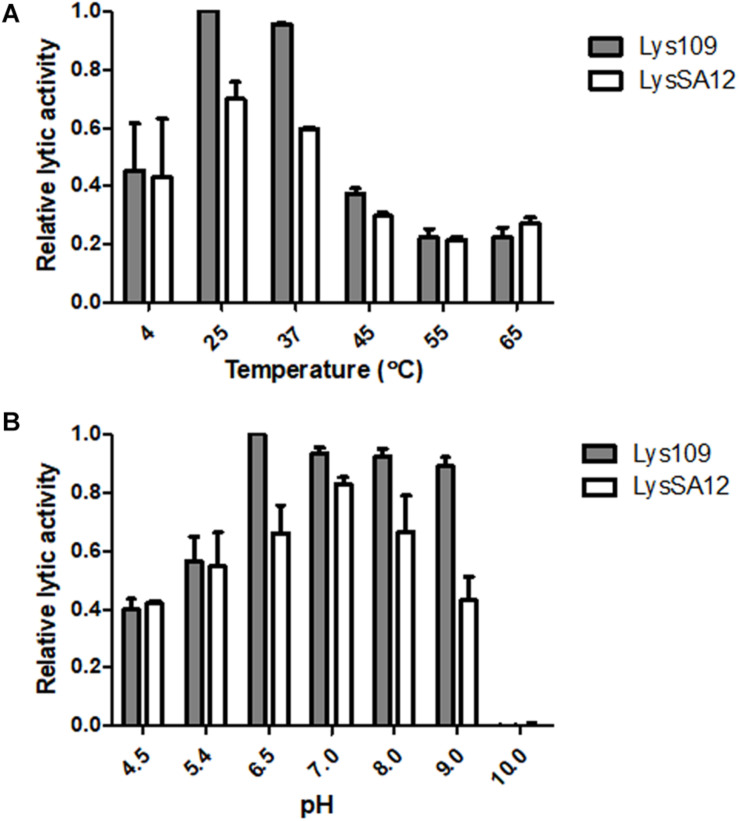
The effects of temperature and pH on the lytic activity of Lys109 and LysSA12. The optimum **(A)** temperature and **(B)** pH of Lys109 and LysSA12 were examined by incubation of the enzymes (300 nM each) with target cells at different temperatures and in different pH buffers, respectively. The relative lytic activities were calculated using the activity of Lys109 assayed in a buffer at pH 6.5 and 25°C, which showed maximal activity.

The thermostability of endolysins was determined (Supplementary Figure S2). Lys109 retained over 95% of its activity after 1 h of incubation at 4 to 37°C, and the lytic activity of Lys109 started to decrease at 45°C. Approximately 40% decrease in its hydrolytic activity was observed at 50°C, and higher temperatures (55 and 65°C) caused complete inactivation of Lys109. LysSA12 showed similar pattern of thermal stability to that of Lys109, indicating that the structural stabilization by the fusion of LysSA12 CHAP to LysSA97 amidase domain plus CBD might not be the reason of the enzymatic improvement of Lys109. Next, the effects of temperature and pH on the lytic activity of Lys109 were evaluated to determine the optimum working conditions of Lys109 (Figures 4A,B). The maximal activity of Lys109 was exhibited at 25–37°C and pH 6.5–9.0. The wide optimum pH range of Lys109 suggest that Lys109 can be used for a wide variety of applications, including foods associated with a high risk for *S. aureus* contamination, such as milk products (Marino et al., 2000) and disinfectants for hospital cleaning (Dancer, 2008).

### Antibacterial Spectrum of Lys109

The antimicrobial activity of Lys109 was examined against staphylococcal strains other Gram-positive and Gram-negative bacteria with different amounts of endolysins, and was compared to that of LysSA12 (Table 1). Lys109 displayed effective lytic activity against all tested staphylococcal strains but not the other Gram-positive and Gram-negative bacteria tested. Although Lys109 has the same antibacterial spectrum as LysSA12, Lys109 showed higher lytic activity against most staphylococcal strains tested compared with LysSA12. These results demonstrated that Lys109 retains the specificity of the original endolysin but has stronger antimicrobial properties.

### Efficacy of Lys109 Against *S. aureus* in Milk

**FIGURE 5:**
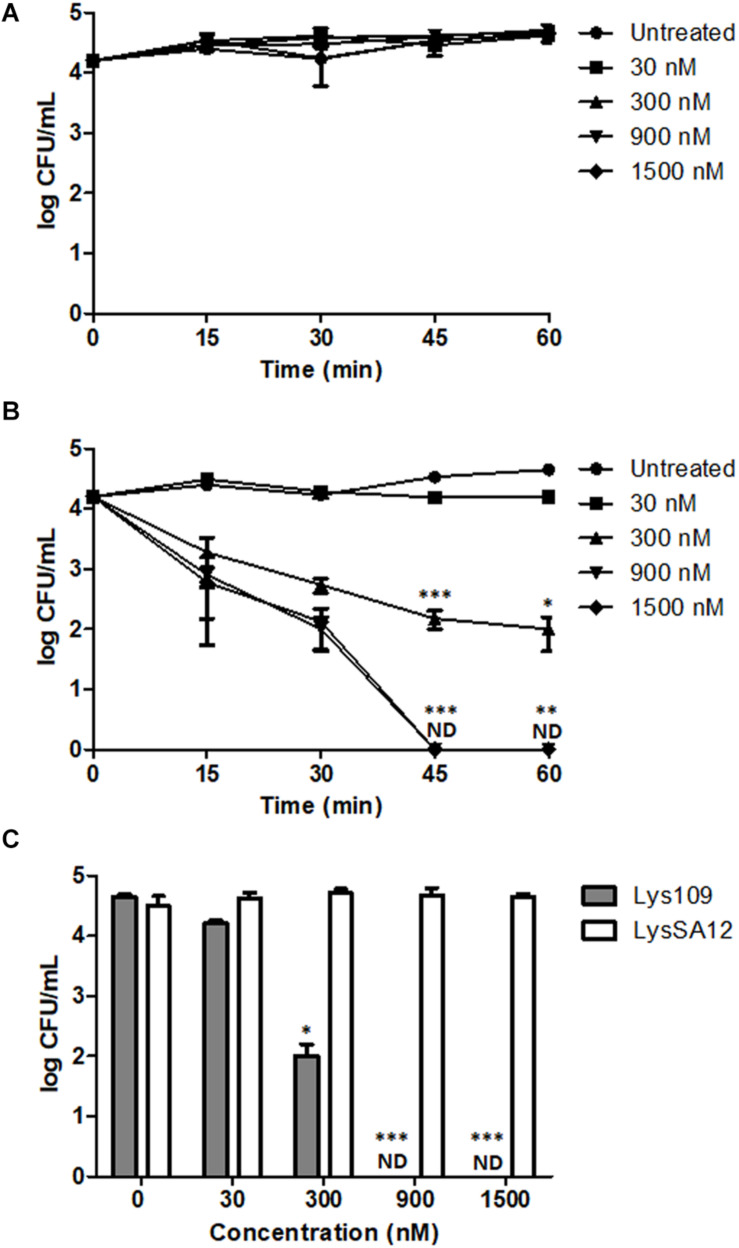
Antibacterial activity of Lys109 and LysSA12 against *S. aureus* CCARM 3090 in milk. **(A)** LysSA12 and **(B)** Lys109 were added to milk artificially contaminated with *S. aureus* CCARM 3090 at different concentrations. Bacterial cells were counted every 15 min for 1 h. **(C)** The number of *S. aureus* CCARM 3090 cells in milk was counted after 1 h of treatment with Lys109 and LysSA12 at concentrations of 0 nM (negative control), 30, 300, 900, and 1500 nM. ND, not detected. Asterisks indicate significant differences (***P < 0.001, **P < 0.01, *P < 0.05).

The comparative antibacterial activity of Lys109 and LysSA12 against a MRSA (*S. aureus* CCARM 3090) strain was examined at various concentrations in milk. Milk was chosen for the test because they have been frequently implicated in staphylococcal foodborne illnesses (Kadariya et al., 2014). In milk artificially contaminated with *S. aureus*, treatment with LysSA12 did not show any reduction in CFU at all tested concentrations even though LysSA12 exerted lytic activity at 100 nM in reaction buffer (Figure 5A), demonstrating the importance of *in vivo* experiments to evaluate the potential of a new antimicrobial. Similarly, LysH5, a LysSA12 homolog, could not kill bacteria cells in milk at 0.15 μM, and only 1-log reduction was observed with 0.8 μM LysH5 (Obeso et al., 2008; García et al., 2010). On the other hand, treatment with 300 nM of Lys109 showed an apparent inhibitory effect on *S. aureus* within 15 min and resulted in a 2-log reduction of bacterial cells after 1h (Figures 5B,C). In addition, the number of *S. aureus* in milk decreased below the detection limit with 900 nM Lys109 within 45 min (Figure 5B), indicating improved antimicrobial activity of the chimeric endolysin compared to parental endolysins. These findings showed that Lys109 has high staphylolytic activity in a complex biomatrix as well as in buffer and suggest that Lys109 has great potential as an antimicrobial agent to control *S. aureus* from dairy products.

### Efficacy of Lys109 Against *S. aureus* on Stainless Steel

**FIGURE 6:**
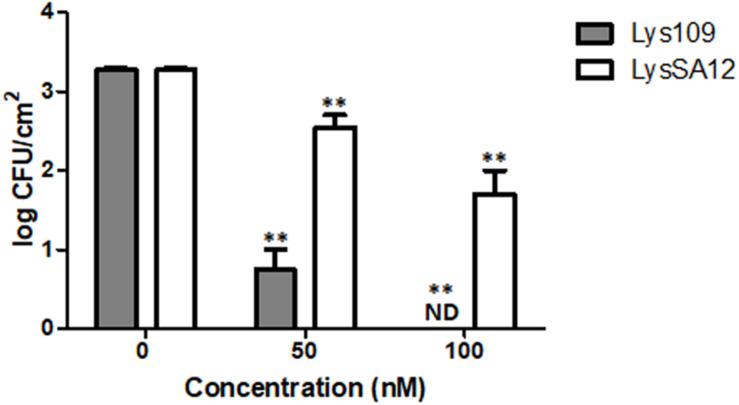
Antibacterial activity of Lys109 and LysSA12 against *S. aureus* CCARM 3090 on stainless steel. The number of *S. aureus* CCARM 3090 cells was counted after treatment with Lys109 and LysSA12 at concentrations of 0 nM (negative control), 50 and 100 nM. ND, not detected. Asterisks indicate significant differences (**P < 0.01).

*Staphylococcus aureus* is a common bacterium encountered in hospital-acquired and device-associated infections (Gwisai et al., 2017). Stainless steel is commonly used for medical devices, and previous reports revealed that MRSA can be viable and grow on it, leading to human infections (Michels et al., 2015; Craft et al., 2019). In this regard, the staphylolytic efficacy of Lys109 was evaluated and compared with its parental endolysin, LysSA12, on stainless steel coupons artificially contaminated with *S. aureus* CCARM 3090. At 100 nM, treatment with Lys109 on the surface of a stainless steel coupon caused the number of bacterial cells to decrease below the detection limit, whereas its parental endolysin was less effective in bacterial cell killing, resulting in only a 1-log bacterial reduction after 1 h (Figure 6). These results suggest the possible use of Lys109 as a disinfectant in hospital settings.

## Discussion

The emergence of multidrug-resistant *S. aureus* has called for novel therapeutic options beyond conventional antibiotics. Endolysins from staphylococcal phages have been proposed as promising alternatives to combat *S. aureus* due to their near species specificity and low probability of developing bacterial resistance (Kashani et al., 2018). In this regard, many researchers have tried to isolate *S. aureus* phages and to identify novel endolysins within the genomes of isolated phages. However, this approach is time-consuming and usually inefficient because staphylococcal endolysins, which are generally classified into five major groups according to their domain architectures (Chang and Ryu, 2017), have more than 90% amino acid sequence identity within each group (Becker et al., 2009b). Besides, poor expression and low solubility levels have further aggravated the situation of getting highly active *S. aureus* endolysins (Manoharadas et al., 2009; Daniel et al., 2010; García et al., 2010). For these reasons, developing a novel *S. aureus*-targeting endolysin with desired properties is of both commercial and academic interest. In this study, we propose an endolysin engineering strategy based on the random domain swapping method to get highly active *S. aureus*-targeting endolysins.

Our screening method involves several noteworthy features as follows. First, we utilized the lytic ability of *S*. Typhimurium phage SPN1S lysRz cassette to lyse *E. coli* cells from within to release expressed endolysins from the cytosol. The SPN1S lysRz cassette consists of an endolysin and two component spanins (Rz and Rz1) which are reported to be essential for disrupting the outer membrane in this final step of host lysis (Rajaure et al., 2015). The co-expression of SPN1S lysRz resulted in strong cell lysis and we were rapidly able to screen highly active chimeric endolysins using 96-well plate format. Second, we constructed two different types of random domain libraries to increase the library diversity, one with variable number of EADs in a random orientation, and the other with only two EADs. A total 19 clones were selected from the libraries based on their strong lytic profiles on *S. aureus* lawns. Sequence analysis revealed that all 19 clones have one or two CHAP domains at the N-terminus and 13 out of the 19 selected clones harbored an amidase domain in the middle of the chimeric endolysins. Consistent with previous reports (Becker et al., 2009a; Schmelcher et al., 2012b; Son et al., 2018), our results indicate that the N-terminal CHAP domain is essential for lysis of *S. aureus* cells and that the amidase domain at the central region may enhance the overall lytic activity of endolysins. Third, the use of heat-killed *S. aureus* cells allowed us to distinguish clearly active chimeric endolysins among clones in the screening process. When *E. coli* and *S. aureus* were co-cultured, it gave rise to false positive clones, some of which showed clear zone on the lawn of *S. aureus* but produced insoluble or inactive form of proteins. Considering that *E. coli* grows faster than *S. aureus* in mixed culture (Fujikawa and Sakha, 2014), we speculate that *E. coli* might inhibit the growth of *S. aureus*, forming inhibition-like zone on the *S. aureus* lawn.

We found that this screening strategy was successful for developing a novel chimeric endolysin and Lys109, which showed the most effective staphylolytic activity, was selected from the random libraries. Lys109 was composed of a LysSA12 CHAP domain, a LysSA97 amidase domain, and a LysSA97 CBD. The efficacy of Lys109 to remove staphylococcal planktonic cells and biofilms was much stronger than that of its parental endolysins, LysSA12 and LysSA97. Then, how Lys109 has superior lytic activity to its parental endolysins? One possible reason is that the increased binding ability of the chimeric endolysin might have led to the improvement of antibacterial activity (Son et al., 2018). Indeed, LysSA97 amidase plus CBD displayed higher binding affinity to the target bacteria than LysSA12 amidase plus CBD (Supplementary Figure S3), suggesting that LysSA97 amidase plus CBD increases the lytic activity of LysSA12 CHAP by enhancing its accessibility to the target bacteria. Alternatively, the peptidoglycan fragment generated by the initial LysSA12 CHAP digestion could be more sensitive to the LysSA97 amidase domain plus CBD than the LysSA12 amidase plus CBD (Becker et al., 2009a). Further structural and biophysical studies of Lys109 will be needed to verify the exact molecular mechanism of enhanced lytic activity provided by domain swapping.

Lys109 showed much stronger antimicrobial activity than its parental endolysin, LysSA12 in milk. The treatment with 300 nM of Lys109 for 1 h showed 2-log reduction of bacterial cells in milk and 900 nM of Lys109 was sufficient to reduce the staphylococcal cells to undetectable levels within 45 min. Several other peptidoglycan hydrolases also have been examined for their antimicrobial activity in milk, but they generally showed low lytic activity in milk (Schmelcher et al., 2015; Verbree et al., 2018). LysH5, a LysSA12 homolog, could not kill bacterial cells in milk with 0.15 μM and only a 1-log reduction was observed with 0.8 μM LysH5 (Obeso et al., 2008; García et al., 2010). The antimicrobial activity of LysSA97, a donor for an amidase and a CBD of Lys109, was also marginal when 1.88 μM of the protein was added in milk (Chang et al., 2017b). More recently, LysSA11 derived from *S. aureus* phage SA11 showed only a 1-log reduction of staphylococcal cell counts in milk when it was added at 2.25 μM for 1 h (Chang et al., 2017a). These results indicate the potential of Lys109 as antimicrobial additives for milk products. Besides, we observed that Lys109 has strong antimicrobial activity on stainless steel. The result demonstrates that stainless steel, the material for medical devices, does not significantly affect the staphylolytic activity of Lys109 and that Lys109 can be possibly used as a disinfectant in clinical settings.

In summary, we proposed an effective screening method to find a novel chimeric endolysin with higher lytic activity and solubility through a random domain swapping of *S. aureus* endolysins. Lys109 was selected from random libraries and showed much stronger lytic activity against staphylococcal strains than its parental endolysins, LysSA12 and LysSA97. Moreover, Lys109 effectively removed staphylococcal biofilms and significantly reduced the number of *S. aureus* cells in milk and on the surface of stainless steel. These results suggest that the random domain swapping method can provide an opportunity for researchers to generate a novel and potent chimeric endolysin with minimal effort. Our strategy therefore holds considerable potential for medical and biotechnological applications to combat multidrug-resistant bacteria such as *S. aureus*.

## Data Availability Statement

The raw data supporting the conclusion of this article will be made available by the authors, without undue reservation.

## Author Contributions

BS and SR conceived and designed the experiments. BS performed the experiments and analyzed the data. YL participated in the experiments. BS and MK wrote the manuscript. SR revised the manuscript. All authors have read and accepted the final manuscript.

## Conflict of Interest

The authors declare that the research was conducted in the absence of any commercial or financial relationships that could be construed as a potential conflict of interest.
