# Supplementary material for: Development of a Novel Chimeric Endolysin, Lys109 With Enhanced Lytic Activity Against Staphylococcus aureus
Source: Front Microbiol. 2021 Jan 15;11:615887. doi: 10.3389/fmicb.2020.615887 (PMC7843465; doi:10.3389/fmicb.2020.615887)
Supplement: Supplementary file 1 [file Data_Sheet_1.pdf]

Supplementary material

Table S1. Plasmids and primers used in this study

Plasmid	Description
pET28a	Kan^r, T7 promoter, His-tagged expression vector
pBAD33	Amp^r, araC, P_{BAD}, pBR322 *ori*
pET28a-LSA12CBD	pET28a with LSA12CBD
pET28a-LSA97CBD	pET28a with LSA97CBD
pET28a-LSA11CBD	pET28a with LSA11CBD
pET28a-LSAP4CBD	pET28a with LSAP4CBD
**Primers**	**Sequence**
EcoR1_SPN1SlysRz_F	AAA GAA TTC GGA GTG AAA CGA TGG ACA TTA ACC AGT TCC GGC
SPN1SlysRz_Sal1_R	TTT GTC GAC TCA CCT GGT CAG CGA ATC GTA C
BamH1_LSA12CHAP_F	AAA GGA TCC ATGC AAG CAA AAC TAA CTA AAA A
LSA12CHAP_BamH1_R	TTT GGA TCC TGA TCG TGG AGC TGT TTC GCT T
LSA12CHAP_Xho1_R	TTT CTC GAG TGA TCG TGG AGC TGT TTC GCT T
BamH1_LSA12AMI_F	AAA GGA TCC GTA CAA TCT CCT ACG CAA GCA
LSA12AMI_BamH1_R	TTT GGA TCC ACT TGA AGC GCT TGA CTC ATT AG
LSA12AMI_Xho1_R	TTT CTC GAG ACT TGA AGC GCT TGA CTC ATT AG
BamH1_LSA97CHAP_F	AAA GGA TCC ATG CCG TCG GTT AGG ACA TAC AG
LSA97CHAP_BamH1_R	TTT GGA TCC TTC TTT TGC GTA GAA TGG ACG GAT
LSA97CHAP_Xho1_R	AAA CTC GAG ATG CCG TCG GTT AGG ACA TAC AG
BamH1_LSA97AMI_F	AAA GGA TCC CAA GAT AAG TTA TCA AAA GGT AAA
LSA97AMI_BamH1_R	TTT GGA TCC ACT ACT TGG CGC ATC AAT TTG TC
LSA97AMI_Xho1_R	TTT CTC GAG ACT ACT TGG CGC ATC AAT TTG TC
BamH1_LSAP4CHAP_F	AAA GGA TCC GGG AAG CAG TTC AAT CCT GAT TT
LSAP4CHAP_BamH1_R	TTT GGA TCC TTT ATC TGG GAA ATT TAA TCT AA
LSAP4CHAP_Xho1_R	TTT CTC GAG TTT ATC TGG GAA ATT TAA TCT AA
BamH1_LSAP4AMI_F	AAA GGA TCC AAA GTA AGT GTT GGA GAT AAA GC
LSAP4AMI_BamH1_R	TTT GGA TCC TTG GTT TTT AGC TGA TGT TTT AAC
LSAP4AMI_Xho1_R	TTT CTC GAG TTG GTT TTT AGC TGA TGT TTT AAC
BamH1_LSA11CHAP_F	AAA GGA TCC ATG AAA GCA TCG ATG ACT AGA AG
LSA11CHAP_BamH1_R	TTT GGA TCC GTC TTT GAA ATT AGG TTC TAT AA
LSA11CHAP_Xho1_R	TTT CTC GAG GTC TTT GAA ATT AGG TTC TAT AA

Table S2. Domain composition of the selected clones

Clone	CHAP	Amidase	CBD
1	SA12		SA11
2	SA12		SA12
3	SA12		SA97
4	SA12	SA12	SA97
5	SA12		SAP4
6	SA12	SA97	SA12
7	SA12	SA97	SA97
8	SA12	SA12	SAP4
9	SA12	SA97	SAP4
10	SA12	SAP4	SA12
11	SA12	SAP4	SAP4
12	SA11	SA12	SAP4
13	SA11		SA12
14	SAP4	SA12	SA12
15	SA11	SA12	SA12
16	SA11		SA11
17	SA12	SA12	SA12
18	SA11	SA12(CHAP)	SA11
19	SA12	SA97	SA11

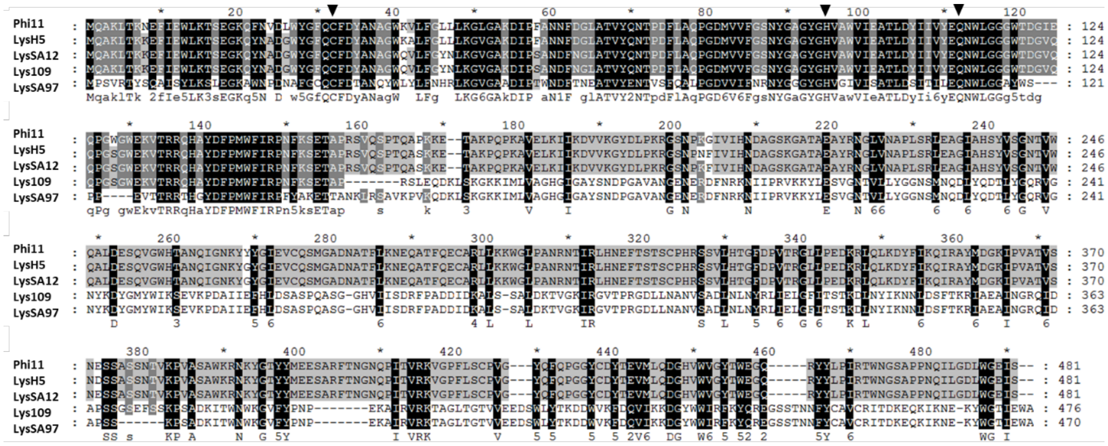

Figure S1. Sequence alignment of Lys109 with other related endolysins. phi11, *S. aureus* phage phi11 endolysin; LysH5, *S. aureus* phage vB_SauS-phiIPLA88 endolysin; LysSA12, *S. aureus* phage SA12 endolysin; LysSA97, *S. aureus* phage SA97 endolysin. Conserved and identical residues are shaded in gray (dark gray, >70% conserved; light gray, >40% conserved) and black, respectively. The conserved Cys-His-Asn triad is indicated by triangles.

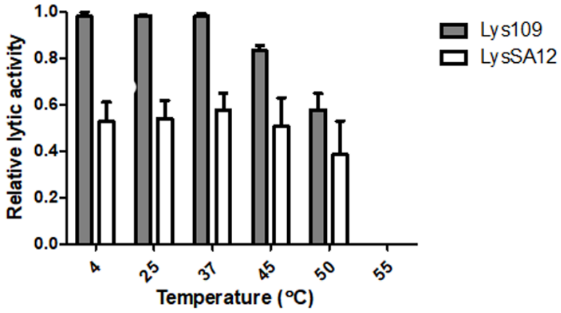

Figure S2. Thermal stability of Lys109 and LysSA12. Enzymes (300 nM each) were incubated at different temperatures for 30 min prior to the lysis assay that was performed against *S. aureus* CCARM 3090 at 25 °C. The relative lytic activities were calculated using the activity of Lys109 stored in a buffer pH 6.5 and at 25 °C, which showed a maximal activity.

A

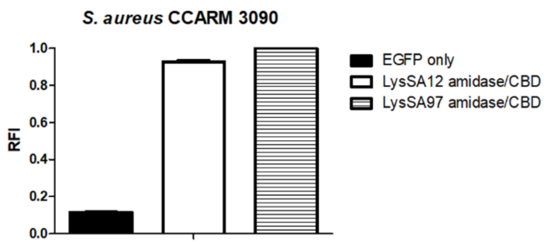

B

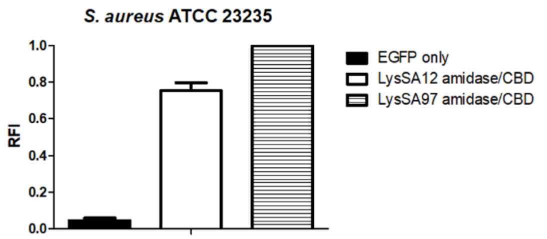

Figure S3. Binding activity comparison among EGFP_LysSA12 amidase domain plus CBD and EGFP_LysSA97 amidase domain plus CBD. The relative binding activity of 1 μM of EGFP_LysSA12 amidase doamain plus CBD and EGFP_LysSA97 amidase domain plus CBD toward **(A)** *S. aureus* CCARM 3090 and **(B)** *S. aureus* ATCC 23235. The binding activity was presented as relative fluorescence intensity (RFI) to the highest measured value. EGFP only did not show binding to *S. aureus*.
